# Disseminated Cryptococcosis Post Eculizumab Therapy: A Case Report and Literature Review

**DOI:** 10.7759/cureus.58852

**Published:** 2024-04-23

**Authors:** Ibrahim Youssef, Muhammad S Abbas, Amir Manafi, Hossein Akhondi, Dima Youssef

**Affiliations:** 1 Internal Medicine, Valley Health System, Las Vegas, USA; 2 Infectious Disease, Valley Health System, Las Vegas, USA

**Keywords:** complement c5, fungal meningitis, myasthenia gravis (mg), cryptococcus neoformans, eculizumab

## Abstract

Eculizumab is a biologic medication used for the treatment of complement-related disorders including anti-acetylcholine receptor antibody-positive generalized myasthenia gravis. It targets C5 complement, preventing its cleavage into active terminal components. Thus, vaccination against encapsulated organisms is advised before starting this treatment. C5 also has a critical role against *Cryptococcus neoformans* infection. Here, we present a case of a 34-year-old man with a history of myasthenia gravis who was treated with prednisone and azathioprine in addition to eculizumab that was added to his regimen about a year ago, and who came to the hospital with headache, and was found to have Cryptococcus meningitis with disseminated cryptococcosis. The patient was negative for human immunodeficiency virus. He was treated with antifungal medications, and his condition improved. Although rarely reported, it is important to have a low threshold for diagnosis of cryptococcosis in patients on eculizumab given its complement inhibition mechanism of action.

## Introduction

Eculizumab is a licensed biologic agent, used for the treatment of atypical hemolytic uremic syndrome, paroxysmal nocturnal hemoglobinuria, neuromyelitis optica spectrum disorder, and anti-acetylcholine receptor antibody-positive generalized myasthenia gravis [1,2]. It is a humanized monoclonal antibody that targets the C5 complement and inhibits its cleavage into active terminal components, C5a and C5b. Inhibition of C5b prevents the formation of the terminal complement and the membrane attack complex, leading to an increased chance of infection with encapsulated organisms [1]. Complement inhibitors carry a markedly increased risk of infection with encapsulated bacteria, especially *Neisseria meningitidis*, exposing individuals to a risk that is up to 2,000 times higher than that of the general population [3]. Following the guidelines outlined by the advisory committee on immunization practices, it is recommended to vaccinate patients against *Neisseria meningitidis* before the initiation of eculizumab therapy [4]. The critical role of C5 against *Cryptococcus neoformans* infection has been shown in previous studies [5], and a limited number of disseminated cryptococcal infections following eculizumab have been reported [6,7]. Here, we present a case of disseminated cryptococcosis after eculizumab treatment.

## Case presentation

A 34-year-old male with a medical history of seropositive myasthenia gravis for the past 13 years presented to the hospital for persistent headache of two weeks duration. The pain was global, 10/10 in intensity, and was associated with neck stiffness, nausea, decreased appetite, and generalized weakness. His myasthenia gravis was managed chronically with prednisone 40 mg two times a day, azathioprine 100 mg once a day, and the latest addition, about a year ago, of eculizumab with a subsequent better myasthenia gravis disease control. The patient was not febrile on presentation and had normal vital signs. His physical exam was remarkable for cervical rigidity. Labs on arrival are shown in Table 1.

**Table 1:** Labs on Arrival to Hospital cells/mcL: cells per microliter, gm/dL: grams per deciliter; mmol/L: millimoles per liter; mg/dL: milligrams per deciliter.

Test	Level	Reference Range
White blood cells	10.50	3.18-12.74 x1,000 cells/mcL
Red blood cells	4.69	4.08-5.80 x1,000,000 cells/mcL
Hemoglobin	15.7	12.3-17.2 gm/dL
Hematocrit	45.0	37.0%-52.0%
Sodium	138	136-145 mmol/L
Potassium	3.4	3.5-5.1 mmol/L
Chloride	102	98-107 mmol/L
Creatinine	1.55	0.7-1.3 mg/dL
Blood urea nitrogen	18	7-18 mg/dL
Blood urea nitrogen/creatinine ratio	12	6-22

**Figure 1:**
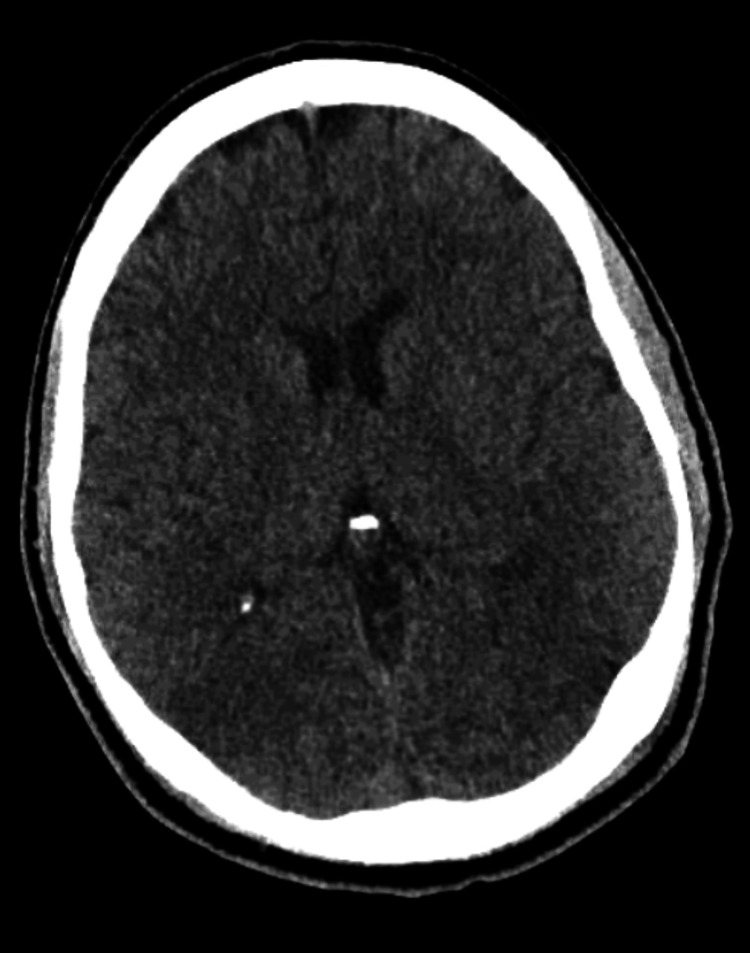
Head CT Head CT without contrast showing a normal brain architecture with no acute intracranial pathology.

**Figure 2:**
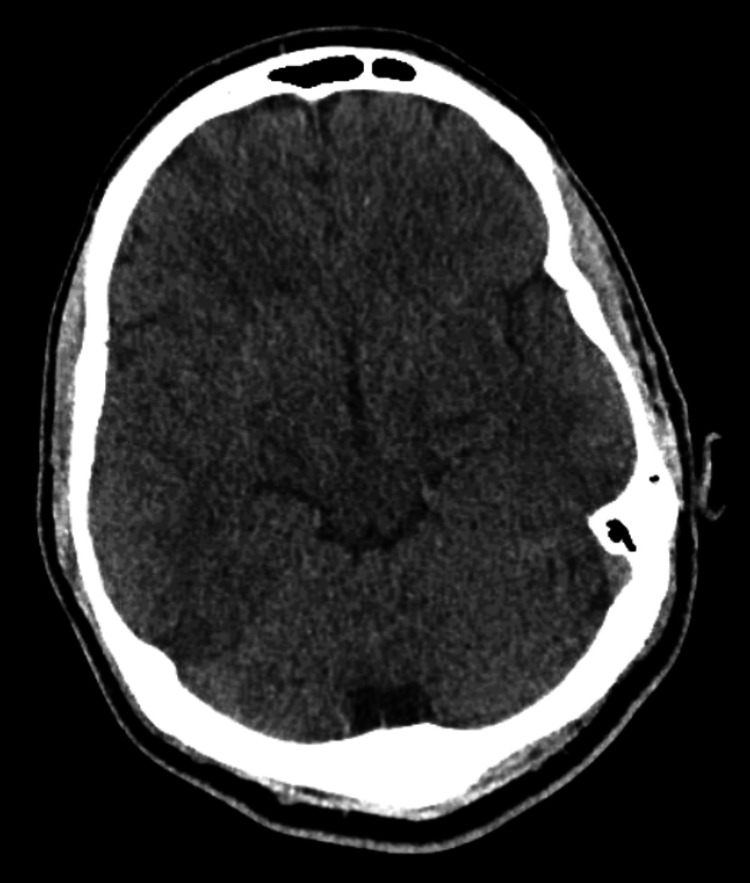
Head CT Head CT without contrast showing a normal brain architecture with no acute intracranial pathology.

**Figure 3:**
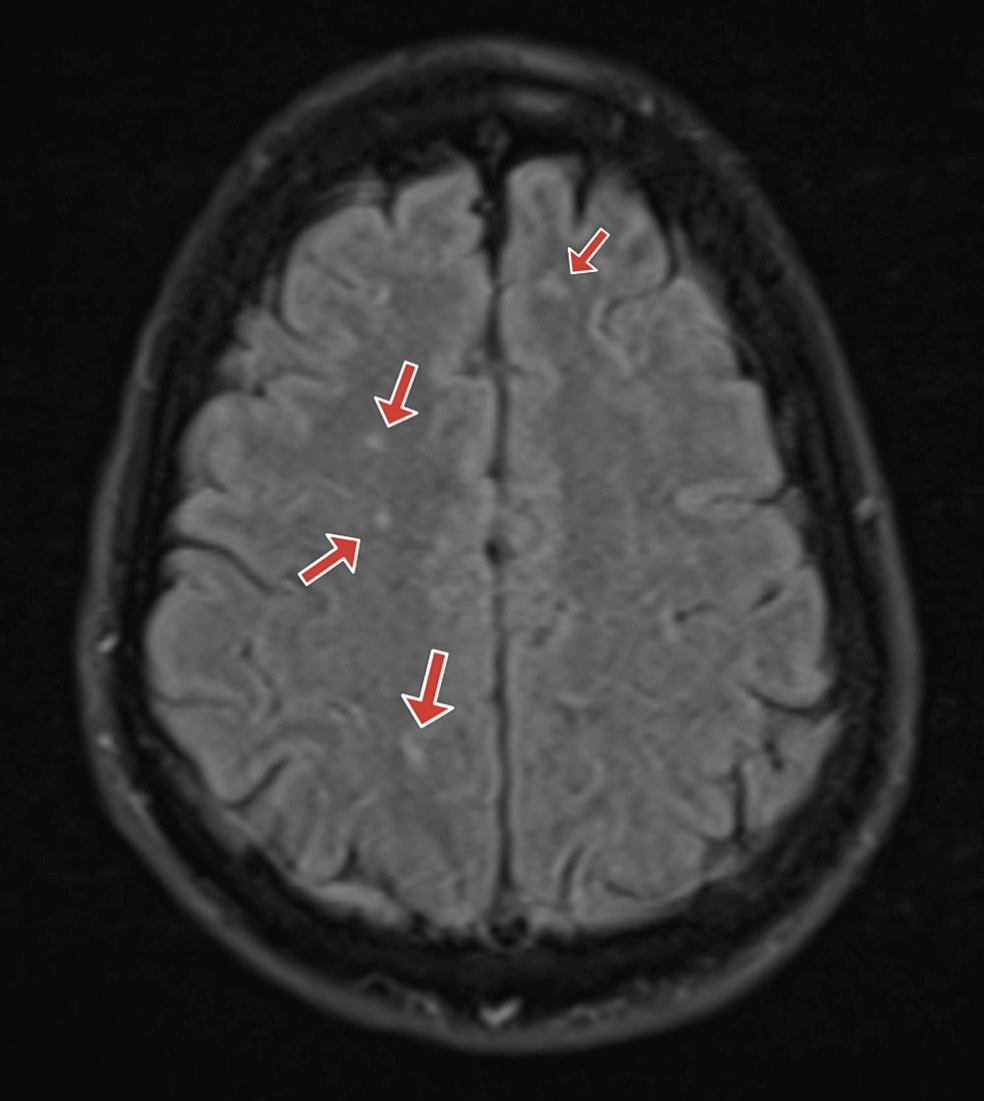
Brain MRI Brain MRI with red arrows pointing at bilateral white matter changes thought to be related to an infection or an inflammation.

**Figure 4:**
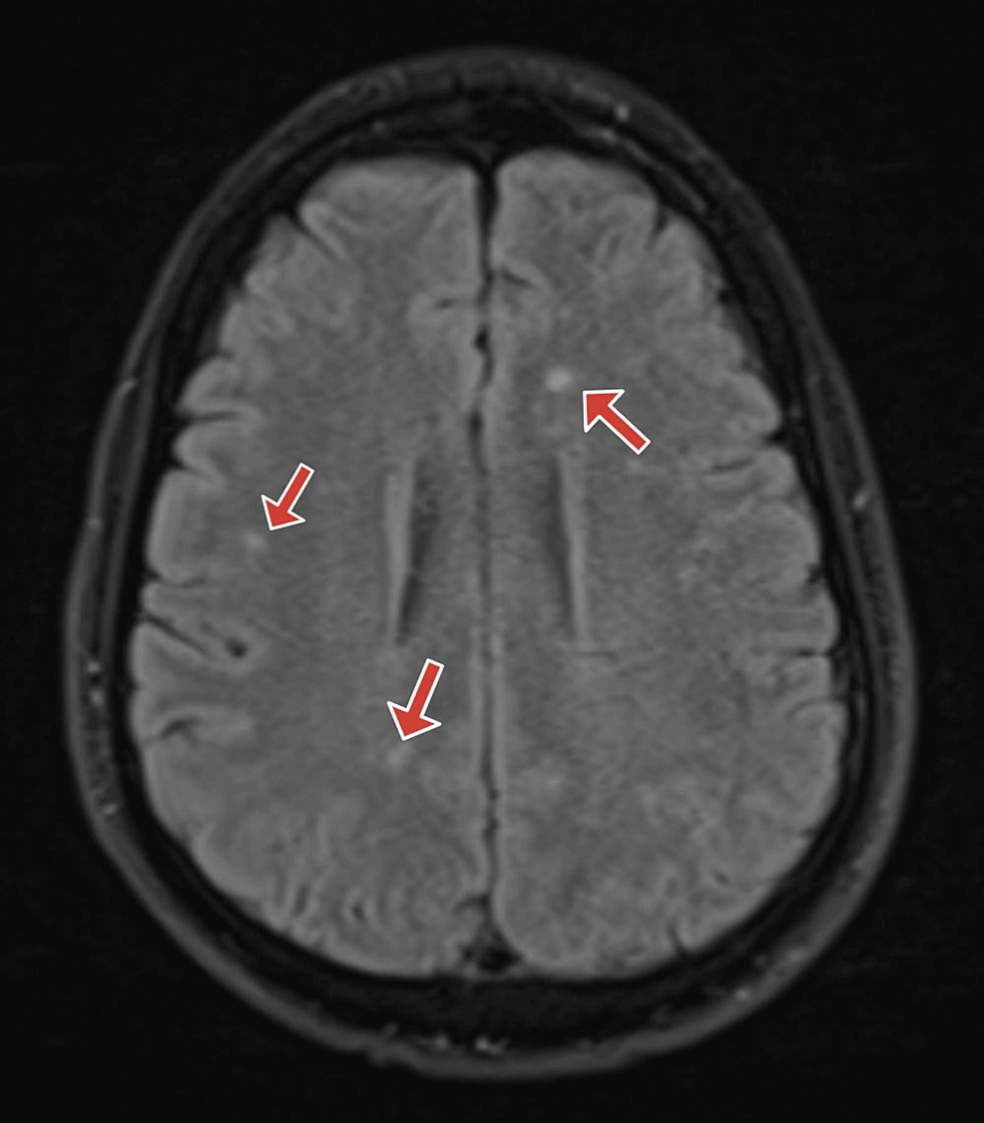
Brain MRI Brain MRI with red arrows pointing at bilateral white matter changes thought to be related to an infection or an inflammation.

**Figure 5:**
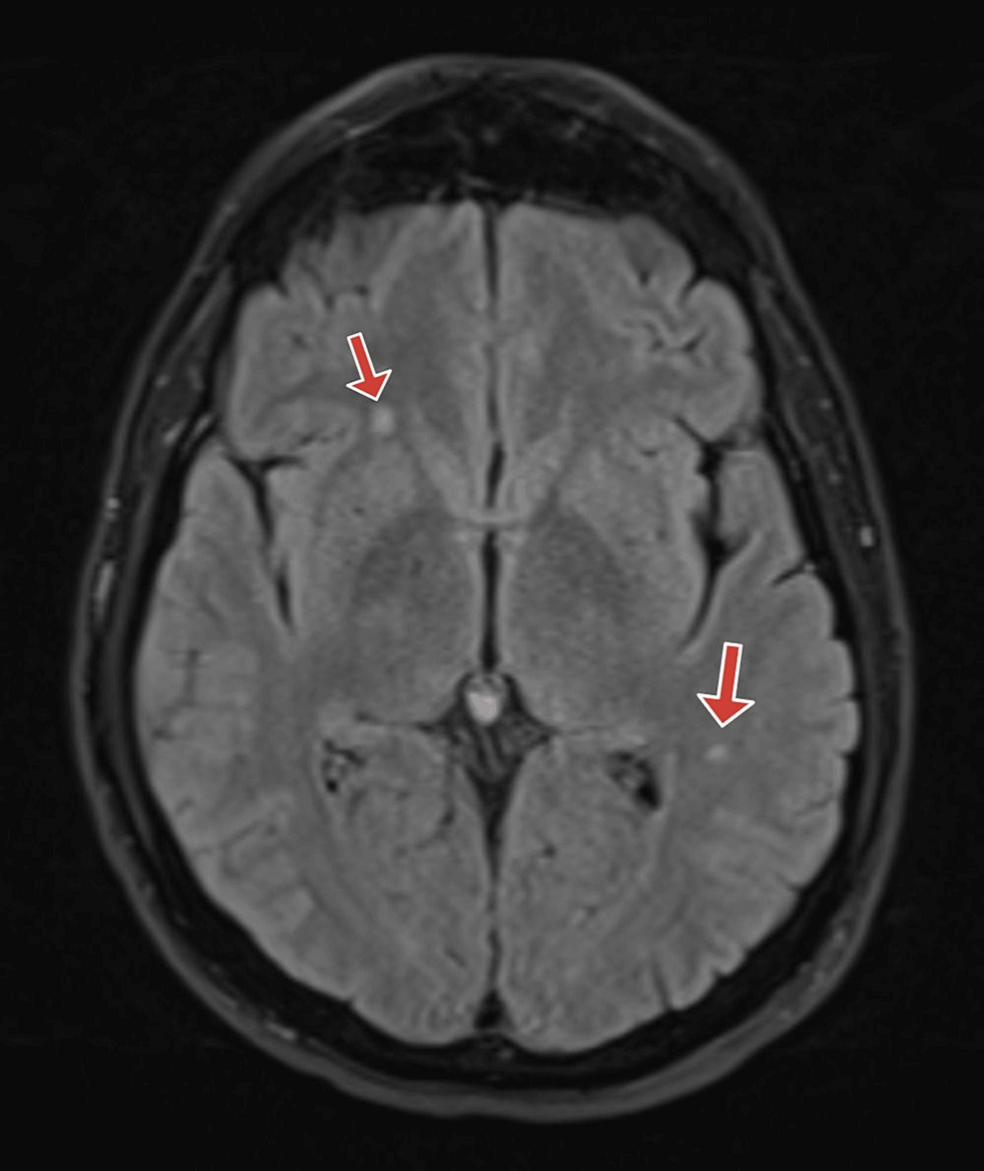
Brain MRI Brain MRI with red arrows pointing at bilateral white matter changes thought to be related to an infection or an inflammation.

**Figure 6:**
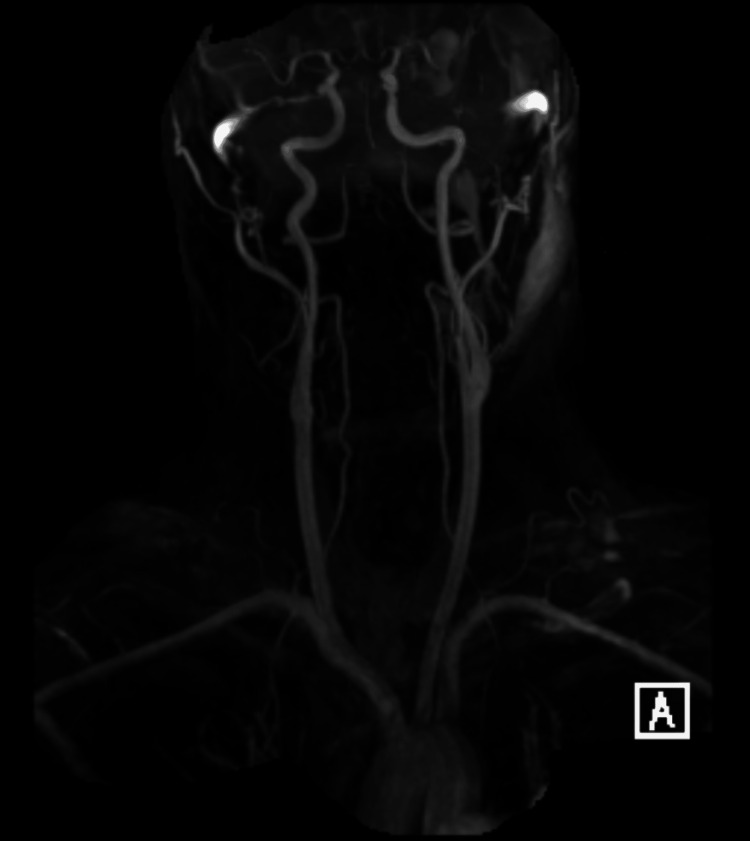
Head and Neck MRA MRA of the head and neck showing a normal vasculature of the head and neck with no significant stenosis, occlusion, dissection, or aneurysms found. MRA: magnetic resonance angiography.

Based on history and physical exam, brain imaging was obtained, and there was a suspicion for a central nervous system infection. Computed tomography (CT) of the head without contrast was obtained and was normal (Figures 1-2). Magnetic resonance imaging (MRI) of the brain was obtained, and showed bilateral white matter changes thought to possibly be due to an infection or an inflammation (Figures 3-5). Magnetic resonance angiography (MRA) of the head and neck was done with no significant stenosis, occlusion, dissection, or aneurysms found (Figure 6).

Given the clinical picture and the imaging results, the patient was started on intravenous (IV) ceftriaxone; a fluoroscopic-guided lumbar puncture was done with an elevated opening pressure at 53 cmH_2_0 and a closing pressure at 18 cmH_2_0. Cerebrospinal fluid analysis is shown in Table 2.

**Table 2:** CSF Cell Count and Chemistry N/A: not applicable; cells/mcL: cells per microliter; mg/dL: milligrams per deciliter; CSF: cerebrospinal fluid.

Test	Tube 1	Reference Range
CSF appearance	Clear	N/A
CSF color	Colorless	N/A
CSF white blood cells	7	0-5 cells/mcL
CSF red blood cells	0	0-1 cells/mcL
CSF neutrophils	1	%
CSF lymphocytes	88	%
CSF monocytes	11	%
CSF glucose	51	40-70 mg/dL
CSF total protein	52	15-45 mg/dL

Cerebrospinal fluid (CSF) polymerase chain reaction (PCR) panel was positive for cryptococcus (Table 3). CSF culture grew yeast.

**Table 3:** CSF PCR Panel CSF: cerebrospinal fluid; PCR: polymerase chain reaction.

CSF PCR	Detection Status
Listeria monocytogenes	Not detected
Streptococcus pneumoniae	Not detected
Neisseria meningitidis	Not detected
Neisseria meningitidis	Not detected
Streptococcus agalactiae	Not detected
Cytomegalovirus (CMV)	Not detected
Enterovirus	Not detected
Herpes simplex virus 2	Not detected
Human herpesvirus 6	Not detected
Human parechovirus	Not detected
Varicella zoster virus (VZV)	Not detected
*Cryptococcus neoformans*/*gattii*	Detected
*Escherichia coli* K1	Not detected

Human immunodeficiency virus (HIV) testing was done and was negative. CD4 count was normal at 483 cells/mcL. Serum cryptococcal antigen was checked and was positive (Table 4).

**Table 4:** HIV, CD4 Count, and Serum Cryptococcus Antigen Testing N/A: not applicable; cells/mcL: cells per microliter; HIV: human immunodeficiency virus; Ab/Ag: antibody/antigen; CD4: cluster of differentiation 4.

Test	Tube 1	Reference Range
Cryptococcus antigen	1:20	Negative <1:5
HIV 1/2 Ab/Ag combo	Non-reactive	N/A
Absolute CD4 count	483	359-1519 cells/mcL

**Figure 7:**
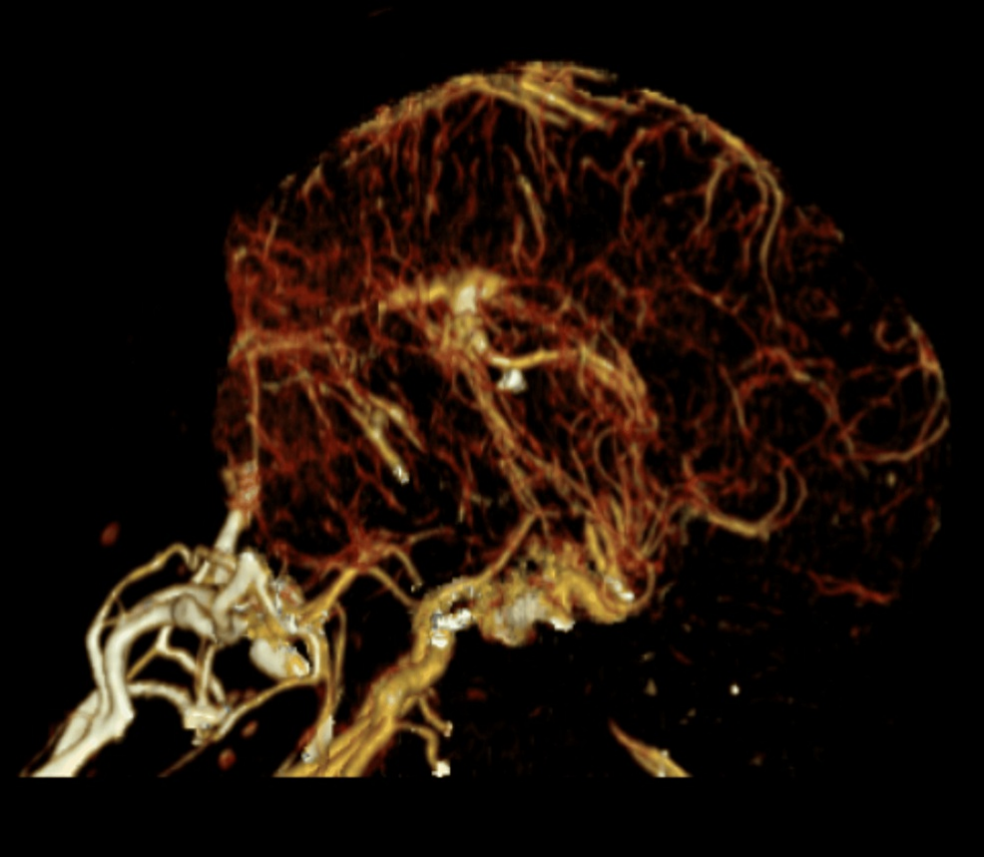
Head CT Venogram CT venogram of the head showing no evidence of dural venous sinus thrombosis.

**Figure 8:**
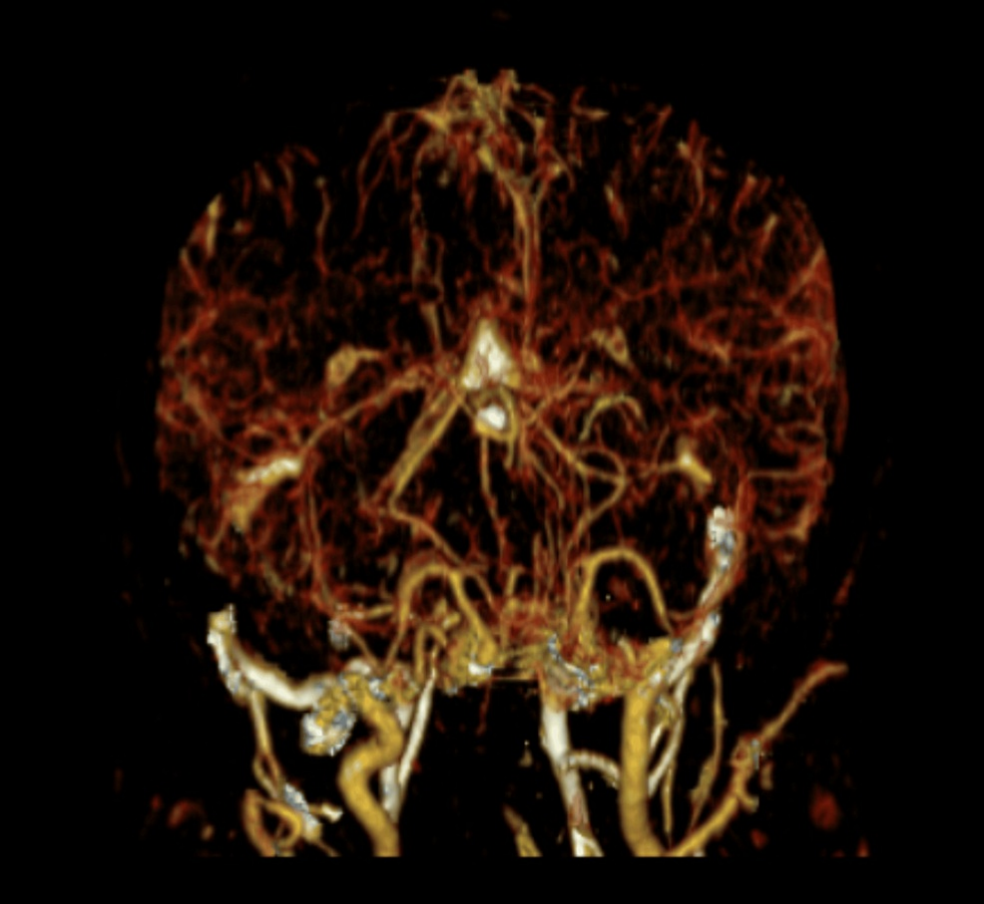
Head CT Venogram CT venogram of the head showing no evidence of dural venous sinus thrombosis.

Eculizumab, azathioprine, and prednisone were held due to the associated risk of immunosuppression. The patient was started on intravenous amphotericin B lipid complex 400 mg daily and oral flucytosine 2,000 mg four times a day. Over the course of the hospital stay, the patient continued to have headache, and two additional therapeutic lumbar puncture procedures were performed. CT venogram of the head was done to rule out dural sinus thrombosis and was negative (Figures 7-8).

The patient’s clinical status improved. He completed two weeks of induction therapy with intravenous amphotericin B lipid complex 400 mg daily and oral flucytosine 2,000 mg every four hours, then was transitioned to oral fluconazole on discharge after the last (third) lumbar puncture procedure was done. The consolidation dose of fluconazole on discharge was 400 mg daily for eight weeks, followed by a maintenance dose of fluconazole of 200 mg daily for at least a year. No immunosuppressive medications were resumed on discharge. The patient followed up at the infectious disease clinic and was found to be doing well, tolerating fluconazole with resolution of his headache. After discharge, prednisone 40 mg twice a day was resumed. The patient continues to be on fluconazole maintenance and is a likely candidate for lifelong suppressive therapy on fluconazole given his underlying myasthenia gravis and need for immunosuppressant treatment.

## Discussion

*Cryptococcus neoformans* and *Cryptococcus gattii* cause most of cryptococcal infections in humans, although other cryptococcal species have occasionally been associated with infection as well. Cryptococcal meningitis occurs after the fungus is contracted by inhalation and then disseminates to the central nervous system [8].

*Cryptococcus neoformans* is a fungus with a polysaccharide capsule [9] that is known to cause central nervous system infections in immunocompromised individuals such as those with HIV, chemotherapy patients, organ transplantation recipients, long-term steroid users, certain hematologic malignancies, or sarcoidosis [10]. The polysaccharide capsule is the most important element of virulence of *Cryptococcus neoformans* [11]. Studies have shown that this capsule activates the complement system in vitro [11] and that complement deficiency can lead to cryptococcal infections [12-14].

We conducted a literature search using PubMed and Google Scholar databases. The search was performed using the keywords "eculizumab", “Cryptococcus”, “cryptococcosis”, and “cryptococcal”. The search aimed to identify any previous case reports of cryptococcal infection associated with the use of eculizumab, and two cases were found [6,7]. To our knowledge, this is the third case in the literature of a patient having a cryptococcal infection after being treated with eculizumab (Table 5).

**Table 5:** Reported Cases of Cryptococcal Infection Post Eculizumab Treatment y/o: year old; HIV: human immunodeficiency virus.

	Authors	Age	Sex	Eculizumab Indication	HIV Status	Other Immunosuppressive Medications	Outcome
Case 1 (2018)	Clancy et al. [6]	23 y/o	Male	Atypical hemolytic uremic syndrome	Negative	None or not mentioned	Death
Case 2 (2023)	Lortholary et al. [7]	58 y/o	Male	Atypical hemolytic uremic syndrome	Not mentioned	None or not mentioned	Treatment with fluconazole
Case 3 (2024)	Youssef et al.	34 y/o	Male	Myasthenia gravis	Negative	Azathioprine and prednisone	Treatment with amphotericin and flucytosine and then with fluconazole

The patients in the two previous cases were adult males [6,7], like in our case. Eculizumab was given to them for the treatment of atypical hemolytic uremic syndrome, while in our case, it was used for the management of myasthenia gravis. HIV testing was negative in one of the previous cases and our case, with no HIV status mentioned in one of the previous cases. No other immunosuppressive medications were mentioned in the other two cases. Our patient was on two other immunosuppressive medications (prednisone and azathioprine) which are known to be implicated in cryptococcal infections in non-HIV patients, but the patient in our case was on them longer than eculizumab which was the latest medication added for myasthenia gravis management. In all three cases, disseminated cryptococcosis occurred with evidence of cryptococcal fungemia. Our patient is the only one with cryptococcal meningitis who had Cryptococcus detected in CSF. In one of the previous cases where the patient passed away, an autopsy confirmed the presence of Cryptococcus.

## Conclusions

Eculizumab inhibits the cleavage of C5 which leads to an increased risk of infection with encapsulated organisms. Vaccination against these organisms is important before initiation of this medication. Due to the role of C5 in fighting Cryptococcus infection, it is important to be on the alert and has a low threshold for diagnosis of cryptococcosis in patients on eculizumab. The co-treatment with other immunosuppressive medications, which can be the case in the management of myasthenia gravis and other autoimmune disorders, may have a role in furthering the risk of cryptococcosis, hence the importance of prompt diagnosis and treatment.
